# Use of Oleuropein and Hydroxytyrosol for Cancer Prevention and Treatment: Considerations about How Bioavailability and Metabolism Impact Their Adoption in Clinical Routine

**DOI:** 10.3390/biomedicines12030502

**Published:** 2024-02-23

**Authors:** Francesco Gervasi, Fanny Pojero

**Affiliations:** 1 Specialistic Oncology Laboratory Unit, ARNAS Hospitals Civico Di Cristina e Benfratelli, 90127 Palermo, Italy; francesco.gervasi1@gmail.com; 2 Department of Biological, Chemical and Pharmaceutical Sciences and Technologies, University of Palermo, 90123 Palermo, Italy

**Keywords:** oleuropein, hydroxytyrosol, cancer, nutrition

## Abstract

The fact that the Mediterranean diet could represent a source of natural compounds with cancer-preventive and therapeutic activity has been the object of great interest, especially with regard to the mechanisms of action of polyphenols found in olive oil and olive leaves. Secoiridoid oleuropein (OLE) and its derivative hydroxytyrosol (3,4-dihydroxyphenylethanol, HT) have demonstrated anti-proliferative properties against a variety of tumors and hematological malignancies both in vivo and in vitro, with measurable effects on cellular redox status, metabolism, and transcriptional activity. With this review, we aim to summarize the most up-to-date information on the potential use of OLE and HT for cancer treatment, making important considerations about OLE and HT bioavailability, OLE- and HT-mediated effects on drug metabolism, and OLE and HT dual activity as both pro- and antioxidants, likely hampering their use in clinical routine. Also, we focus on the details available on the effects of nutritionally relevant concentrations of OLE and HT on cell viability, redox homeostasis, and inflammation in order to evaluate if both compounds could be considered cancer-preventive agents or new potential chemotherapy drugs whenever their only source is represented by diet.

## 1. Introduction

Cancer insurgence and progression are complex processes, depending on the combination of unmodifiable genetic and modifiable environmental/lifestyle-related factors. With this premise, it sounds perfectly understandable that scientific evidence has corroborated the role of a healthy diet and dietary intervention as potentially beneficial approaches contributing to cancer prevention [1,2,3]. Epidemiological and experimental evidence has confirmed that the so-called Mediterranean diet is a source of molecules that may mitigate cancer risk factors like chronic inflammation and redox imbalance, thus participating in the prevention of carcinogenesis in terms of loss of cell cycle regulation and proper immune modulation, as well as in the inhibition of angiogenesis and metastasis. Moreover, some of these natural compounds may have a cytotoxic effect, making them interesting alternatives to or candidates for integration into conventional therapeutic approaches [2,4,5,6,7,8,9].

**Figure 1:**
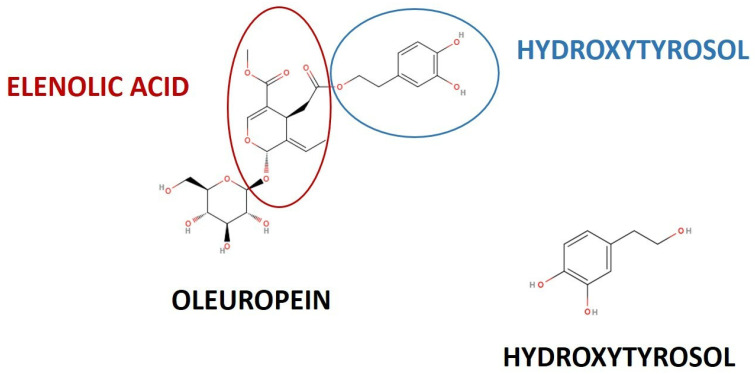
Molecular structure of OLE and HT. As it can be appreciated from the image, OLE structure is characterized by the presence of an ester bond between elenolic acid and HT (thus, HT may be released as an OLE decomposition product). Molecular structures were designed with MolView Copyright© 2014–2023 Herman Bergwerf available at https://molview.org/ (accessed on 13 February 2024).

Among Mediterranean diet phenols, secoiridoid oleuropein (OLE) is the most abundant phenolic compound in *Olea europaea* L. tree leaves (OLE content up to 14–19% in olive leaves), followed by its degradation derivative hydroxytyrosol (3,4-dihydroxyphenylethanol, HT, 2.28 mg/g of olive leaf extract) (Figure 1) [10,11,12,13,14,15,16].

OLE and HT are also found in the fruit of *Olea europaea* L. and in olive oil; thus, they are easily ingested as part of a routine diet, but they can also be obtained from other sources, e.g., olive mill wastewater [16,17,18,19,20]. Both compounds have attracted attention for their accessibility, safe profile, powerful antioxidant and scavenging activity against reactive oxygen species (ROS), and controversial anti-inflammatory action. For more than two decades, OLE and HT (together or alone) have been the focus of intense research efforts in the context of infectious diseases and prevention/management of chronic non-communicable diseases, including cancer, with encouraging results from in vitro and in vivo models [19,20,21,22,23,24,25,26].

On this basis, it would be difficult to understand the reasons behind the lack of systematic testing of OLE and HT as supplements to prevent the insurgence of cancer or support the management of hematological malignancies and solid tumors. This becomes clearer considering that, despite promising proof in the field, experimental evidence about OLE and HT bioavailability in humans and animals clearly demonstrates that OLE and HT act as cancer-preventive agents and cytotoxic drugs mainly at concentrations far from plasma levels reachable through nutrition, an aspect often interpreted as marginal that we discuss in detail in this review.

Also, as explained in the following paragraphs, the complexity and diversity of molecular mechanisms resulting in net OLE and HT action has led to questions regarding the possibility that these compounds might even facilitate the expansion of neoplastic clones at nutritionally relevant concentrations.

In this review, we discuss the available data on the use of OLE and HT as anti-cancer drugs and the feasibility of their application in the context of clinical routine now or in the near future.

## 2. Effects of OLE and HT on Solid Tumors

OLE and HT activity against solid tumor insurgence and development has been challenged in a large number of experimental models, both in vivo and in vitro. No single shared molecular mechanism and/or triggered cellular response seems implicated in OLE and HT cytotoxicity, resulting in an articulated frame that imposes a separate dissertation for every type of studied solid tumor. To facilitate critical interpretation, in each subsection, OLE and HT assayed doses are indicated, with the half-maximal inhibitory concentration (IC50) and the half-maximal effective concentration (EC50) reported as exact values, mean ± standard deviation (S.D.), or mean ± standard error of the mean (S.E.M.) whenever provided by the authors. A list of experimental models used to study OLE and HT cytotoxicity in cancer cells is reported in Table 1.

### 2.1. Skin Carcinogenesis and Melanoma

Malignant melanoma is a malignancy arising from the transformation of melanocytes, with increasing incidence worldwide. On the basis of the tissue where the primary lesion appears, four major subtypes can be distinguished: cutaneous melanoma (non-glabrous skin), acral melanoma (glabrous skin), mucosal melanoma (melanocytes in the mucosal tissues), and uveal melanoma (uveal tract of the eye). Among these major subtypes, it is possible to distinguish some particular variants: amelanotic/hypomelanotic melanoma, a subtype of cutaneous melanoma with low or absent melanin; desmoplastic melanoma, a spindle cell tumor exhibiting signs of dense scar-like fibrosis; spitzoid melanoma, sharing histopathological characteristics with Spitz nevi; acral lentiginous melanoma, with a lentiginous growth pattern [106,107,108,109].

OLE seems to be effective in the prevention of skin carcinogenesis in vivo. Orally administered 10 mg/kg and 25 mg/kg OLE reduced skin carcinogenesis (expressed in terms of number of tumors per mouse) in UVB-irradiated albino hairless HOS: HR-1 mice [27]. For 25 mg/kg OLE, this effect was associated with a persistent reduction in (I) the total volume of tumors per mouse, (II) the expression levels of invasion enzymes matrix metalloproteinase 2 (MMP2), pro-MMP9, and MMP9, (III) tissue angiogenesis marker vascular endothelial growth factor (VEGF) and cyclooxygenase-2 (COX-2) levels, and (IV) the percentage of skin Ki-67+ cells and platelet endothelial cell adhesion molecule-1 (PECAM-1 or CD31)+ areas [27,110,111,112,113]. OLE also seems effective in the prevention of melanoma growth and metastasis. In an in vivo B16F10 (mouse melanoma cell line) allograft model of high-fat diet (HFD)-induced melanoma progression in C57BL/6N mice, HFD containing 0.02% and 0.04% OLE reduced HFD-driven tumor growth and lymph node metastasis, with a mechanism involving (I) inhibition of cell proliferation, as indicated by the reduction in the percentage of cells positive for proliferation markers Ki67, Cyclin D1, and cyclin-dependent kinase 4 (CDK4) cells, (II) suppression of angiogenesis (reduction in CD31, VE-cadherin expression, VEGF-A, VEGF-C, VEGF-D, VEGF receptor 2 -VEGFR2- and VEGFR3), and (III) inhibition of lymphangiogenesis, as proved by staining for lymphatic vessel endothelial hyaluronan receptor (LYVE-1). According to further in vitro experimental assays, OLE anti-angiogenetic and lymphangiogenetic action relies on the inhibition of lipid and M2-macrophage accumulation [28,113,114,115].

In vitro results for OLE are conflicting. Incubation of human amelanotic melanoma cell line C32 with 100 μM, 400 μM, and 1000 μM OLE for 72 h promoted cell viability [29]. On the contrary, incubation of human melanoma cell lines A375, WM266-4, and M21 with 250 μM, 500 μM, and 800 μM OLE for 72 h produced a dose-dependent decrease in cell viability. As deepened in A375 cells, an increase in OLE effectiveness with time may be detected. OLE induced inhibition of cell viability after 24 h at a concentration of 800 μM, whereas after 48 and 72 h, a decrease in the percentage of viable cells became significant with a concentration as low as 250 μM [30]. After 48 h treatment, 500 μM OLE increased the rate of apoptosis in A375 cells. In addition, 24 h incubation with 250 μM OLE was sufficient to reduce invasiveness of A375 cells, while 48 h incubation with the same OLE concentration reduced the phosphorylation of pro-survival kinase Akt [30,116].

As regards in vitro evidence for HT, the effects on cell growth seem to be cell line-dependent. Treatment of C32 cells with 100 μM HT for 72 h increased cell viability, which was instead reduced by incubation with 400 μM and 1000 μM HT [29]. Incubation of human melanoma cell line A375 with 100 μM and 200 μM HT significantly diminished cell viability after 48 h. On the contrary, 48 h treatment of melanoma cell line MNT1 with 100 μM and 200 μM HT had no significant effect on cell viability. The authors attributed this dissimilarity between the mentioned cell lines to differences in the active metabolic pathways. Expression analysis revealed a significant transcriptional upregulation of lactate dehydrogenase B (LDHB) and LDHC (accounting for lactate conversion into pyruvate) and glutamine synthetase (GLUL) in MNT1 cells in comparison with the A375 cell line, while sodium-coupled neutral amino acid transporter 1 (SNAT1) and SNAT2 (involved in glutamine transport within the cell), monocarboxylate transporter 4 (MCT4, accounting for lactate export), glycolytic enzyme glucose-6-phosphate dehydrogenase (G6PD), and excitatory amino acid transporter 3 (EEAT3) were downregulated in MNT1 cells vs. A375 [31,117,118,119,120,121,122,123,124]. In a further report, treatment of human melanoma cell lines A375, HT-144, and M74 with 50–250 μM HT produced a dose- and time-dependent decrease in cell viability after 24, 48, and 72 h [32]. A detailed analysis on A375 cells (treated with 250 μM, 375 μM, and 500 μM HT) and HT-144 cells (incubated with 250 μM, 350 μM, and 450 μM HT) performed for 24 and 48 h revealed an increase in the rate of apoptosis in both cell lines, with a dose- and time-dependent increase in tumor suppressor p53 and reduction in growth-promoting kinase Akt protein levels [32,125]. The activation of apoptosis pathway was further confirmed by an increase in apoptosis markers pro-activated and cleaved (activated) forms of caspase-3, a dose- and time-dependent increase in cleavage (activation) of poly ADP-ribose polymerase 1 (PARP-1), and a dose-dependent increase in the phosphorylation of histone H2AX (γH2AX) [32,126,127,128,129]. HT-mediated induction of apoptosis was related to ROS accumulation in both cell lines at the indicated HT concentrations, which was detected after 24 and 48 h [32,130].

### 2.2. Thyroid Cancer

Thyroid cancer is a category of neoplastic lesions with a highly variable degree of aggressiveness, arising from parafollicular C cells (resulting in medullary thyroid cancers) and follicular thyroid cells (producing follicular thyroid cancer, papillary thyroid cancer, poorly differentiated thyroid cancer, Hürthle cell cancers, and anaplastic thyroid cancer) [131,132,133].

In vitro, treatment of human papillary thyroid carcinoma cell line TPC-1 and poorly differentiated thyroid gland carcinoma cell line BCPAP with 50–100 μM OLE for 48 h produced a significant reduction in cell viability attributable to S phase and G2/M phase cell cycle block, respectively. In both cell lines, 50–100 μM OLE exerted an antioxidant activity against hydrogen peroxide (H_2_O_2_)-induced perturbation of ROS homeostasis. Also, 100 μM OLE caused a short-lasting (30 to 60 min) reduction in phosphorylated forms pro-survival kinases ERK (phospho-ERK) and Akt (phospho-Akt) in TPC-1 and BCPAP cells [33,134].

Incubation of papillary thyroid cancer cell lines TPC-1 and FB-2 with 324–973 μM HT decreased cell viability after 24 and 48 h in a dose-dependent manner. A stronger action was exerted on follicular thyroid cancer cell line WRO, whose cell viability was reduced even at lower doses of HT after 24 h (162 μM) and 48 h (65 μM) treatment [34]. After 24 h incubation, 324 μM HT elicited an increase in the percentage of apoptotic and necrotic cells in all the three mentioned cell lines (with a concomitant downregulation of pro-proliferative cyclin D1 and upregulation of tumor suppressor p21^WAF1/Cip1^ at both mRNA and protein levels), increased protein level of tumor suppressor p53, and activated the intrinsic pathway of apoptosis, as corroborated by the increase in cleaved PARP and cleaved caspase-3 levels, Bcl-2-associated agonist of cell death (Bad) and caspase-9 protein levels, and the release of mitochondrial cytochrome c [34,114,126,127,135].

### 2.3. Lung and Pleural Cancers

Currently, lung cancer represents the most commonly diagnosed cancer and the main cause of cancer-related deaths worldwide, including small cell carcinoma and more common non-small cell carcinoma [136,137].

In adenocarcinomic human alveolar epithelial cells A549 (a model for non-small cell lung cancer), 50 μM and 150 μM OLE-induced apoptosis after 24 h incubation was mediated by the decrement in Bcl-2 and Bcl-XL anti-apoptotic proteins flanked by the increase in (I) mitochondrial-located pro-apoptotic protein Bax, (II) cytochrome c release from the mitochondria, (III) activation of apoptosome component apoptotic protease activating factor-1 (Apaf-1), (IV) activation of caspase-3, and (V) mitochondrial methylglyoxal detoxicating enzyme Glo2 (mGlo2), which physically interacted with Bax [35,126,127,138]. Consistent with these data, incubation of non-small cell lung cancer cell line H1299 with 50–200 μM OLE for 24 h elicited a dose-dependent G2/M phase cell cycle block and apoptosis, with effects on Bcl-2, Bax, cytochrome c, and caspase-3 that were similar to those documented for A549 cells [36]. However, the underlying molecular mechanism ruling OLE activity differed between the two cell lines. The effects elicited by 150 μM OLE on mGlo2 levels in A549 cells were strictly dependent on OLE-induced increase in superoxide dismutase 2 (SOD2) detoxicating action against superoxide (O_2_^.-^), and on the inhibition of the Akt signalling pathway. This was not surprising, since in A549 cells, O_2_^.-^ supports Akt activations, promoting cell survival [35]. In H1299 cells, the observed apoptosis was instead determined by OLE-induced phosphorylation of p38 mitogen-activated protein kinase (MAPK), accompanied by an increased rate of phosphorylation of activating transcription factor-2 (ATF-2), involved in cell cycle regulation, as documented in both tumorigenesis and cell death, and the upregulation of genes ruling cell metabolism and apoptosis [36,139,140,141,142,143].

In A549 cell line, HT showed an increase in effectiveness of its anti-proliferative activity with respect to time, with the IC50 values changing from 230.60 μM to 149.36 μM in 72 h [37]. This piece of data was confirmed in another report. In fact, for A549 cells, mean IC50 ± S.E.M. = 147.0 ± 16.5 μM was reported after 48 h incubation [38].

Malignant pleural mesothelioma arises from mesothelial cells and is characterized by a pronounced aggressiveness and poor prognosis. On the basis of histological features, mesothelioma is classified as epithelioid, sarcomatoid, or biphasic, with epithelioid type offering the best median survival [144]. Pleural epithelioid mesothelioma REN cell line was utilized to demonstrate that OLE exhibited a cytotoxic activity (IC50 = 25 μg/mL, ≈46 μM), and that both OLE and HT (10–100 μM) mobilized extracellular Ca^2+^ in a dose-dependent manner [39].

### 2.4. Breast Cancer

Breast cancer is the most frequent malignant tumor in women worldwide, with a constantly rising incidence [145,146]. Treatment of breast cancer is based on the molecular subtype, a classification that in the first instance takes into account the immunohistochemically assayed expression of hormone receptors estrogen receptor (ER) and progesterone receptor (PR), and gene amplification or overexpression of human epidermal growth factor receptor 2 (HER2). Triple negative breast cancer identifies a category of tumors lacking the three mentioned receptors [146].

In an in vivo model of tumor xenograft (triple negative MDA-MB-231 cell line) in BALB/c OlaHsd-foxn1 mice, animals receiving 50 mg/kg OLE for 4 weeks showed a decrease in tumor size, together with a reduction in actors involved in cell growth/proliferation: transcription factor NF-κB and cyclin D1 [40,147,148]. Instead, levels of tumor suppressor p21^WAF1/Cip1^ increased after OLE injection. The effects elicited by OLE were accompanied by the induction of apoptosis, as demonstrated by caspase-3 activation, increase in Bax levels, and reduction in Bcl-2 protein expression [40].

In vitro, OLE action appears to be independent of HER2 gene amplification/overexpression and hormone receptor status. Using MCF-7 cell line, which is devoid of HER-2 overexpression [149,150], 200 μg/mL (≈370 μM) OLE for 48 h showed a specific cytotoxic effect on MCF-7 cell line, leaving the non-cancerous cell line MCF-10A unharmed. The observed effect on cell viability was accompanied by the upregulation of the expression of *Prdx1-Prdx6*, encoding for antioxidant and chaperone proteins peredoxins [41,151,152,153,154]. The induction of apoptosis triggered by the same concentration of OLE in MCF-7 cells was confirmed in another study after 12 h incubation [42]. Treatment of MCF-7 cells with 200 μM and 400 μM OLE for 24 h produced a notable decrease in cell viability. Specifically, at a concentration of 400 μM, OLE increased MCF-7 cell death by apoptosis induction [43]. OLE effects might depend on the upregulation of p53 and Bax, and downregulation of Bcl-2, as demonstrated by incubating MCF-7 cells with 200 μM OLE for 48 h [44]. Other studies using a very large concentration of OLE (600 μg/mL, ≈1100 μM) shed some light on a different mechanism ruling OLE-dependent induction of apoptosis. In fact, treatment of MCF-7 cells with the mentioned OLE concentration for 48 and 72 h reduced histone deacetylase 2 (HDAC2) and HDAC3 gene transcription in a time-dependent manner [49,155,156], and downregulated oncomiRs miR-21 and miR-155 [48,157,158].

However, OLE-mediated effects on ER-positive cell viability may also be appreciated at lower doses. In fact, 30 μM and 50 μM OLE were able to reduce MCF-7 cell viability after 48 h, with no significant increase in the apoptotic rate [46]. Moreover, 150 μM OLE reduced cell viability of ER-positive MCF-7 and T47D cells after 24 h [47]. Further experiments on MCF-7 cell line demonstrated a dose-dependent OLE-mediated antiproliferative effect on 17β-estradiol (E2)-induced cell growth when OLE concentration was used in the range of 10–75 μM. Instead, concentrations ≥ 100 μM were found to be toxic [45]. As regards a possible anti-estrogenic action, 10 μM OLE had no effect on estrogen receptor α (ERα) basal activation, and 10–75 μM OLE had irrelevant activity on E2-induced ERα activation and E2-modulated ERα expression, but reduced E2-induced ERK1/2 phosphorylation [45]. In ER-positive cells, invasiveness may be suppressed by OLE-mediated induction of autophagy. In fact, treatment of MCF-7 and T47D cell lines with 100 μM OLE reversed hepatocyte growth factor (HGF)- and 3-methyladenine (3-MA, an autophagy inhibitor)-induced migration, upregulating LC3II/LC3I and Beclin1, while downregulating p62 [47,159,160,161].

OLE was also able to reduce proliferation in triple negative breast cancer cell lines MDA-MB-231 and MDA-MB-468 after 48 h incubation (IC50 = 500 μM), with RNA sequencing revealing alterations of the expression profile of genes involved in cell death, apoptosis, and response to stress [50]. Other reports documented that a dose as low as 50 μM was able to reduce MDA-MB-231 cell viability after 72 h incubation [46] and IC50 = 36.2 μM was estimated for 72 h treatment [51]. A further attempt to deepen the mechanism of action in triple negative breast cancer showed that 12.5–100 μM OLE affected MDA-MB-231 cell viability in a dose- and time-dependent manner, reducing cellular migration and invasion capabilities, especially at doses ≥ 25 μM. OLE was able to induce dose-dependent apoptosis after 72 h incubation. A deeper analysis, performed using a dose of 100 μM, revealed caspase-3/7 activation after 48 and 72 h, and a reduction in NF-κB phospho-p65 nuclear localization after 12 h [51,126,127,147,148]. In a breast cancer cell line identified by the authors only by the letters “MDA”, 200 μg/mL OLE was able to interfere with the metastatic process, causing a time-dependent increase in mRNA levels of MMP inhibitors TIMP metallopeptidase inhibitor 1 (TIMP1) and TIMP3, while TIMP4 showed no further increase after 48 h incubation. Simultaneously, the same concentration of OLE triggered a time-dependent decline in MMP2 and MMP9 mRNA levels [52,110].

As regards HT activity in vivo, in a model of dimethylbenz[α]anthracene-induced mammary tumors in Sprague–Dawley rats treated with HT (0.5 mg/kg, 5 days/week for 6 weeks) reduced tumor growth, modulating the expression of genes involved in apoptosis and cell proliferation/survival [53].

The relation between HT cytotoxic action in vitro and HER2 overexpression/hormone receptor status is less clear. Incubation of MCF-7 cell line with 50 μg/mL HT (≈324 μM) for 12 h was sufficient to trigger apoptosis [42], despite the fact that this result was not uniformly reproduced. In other experimental settings involving MCF-7 cells, the anti-proliferative activity of HT became evident at higher as well as even lower concentrations. HT used in the range of 5–400 μM for 16 h had no effect on MCF-7 cell proliferation; it became inhibited only at 600 μM [54]. Another report documented a dose- and time-dependent effect of HT on MCF-7 cell viability, with 250 μM decreasing the percentage of viable cells after 72 h treatment, and 400 μM HT reducing cell viability as a consequence of 48 and 72 h incubation [46]. However, at sub-lethal concentration (200 μM), HT had an impact on both (I) oxidative stress response, augmenting mRNA and protein levels of transcription factor nuclear respiratory factor 2 (Nrf2) and upregulating the transcription of its targets glutathione S-transferase alpha 2 (GSTA2) and heme oxigenase-1 (HO-1), and (II) energy homeostasis, reducing mRNA level of mitochondria biogenesis regulator PPARγ coactivator-1α (PGC-1α), while increasing its protein level and downregulating expression (in terms of mRNA) of mitochondrial function regulators estrogen-related receptor α (ERRα) and deacetylase sirtuin 3 (SIRT3) [54,162,163,164,165,166,167,168,169]. Other research records showed that a reduction in ER-positive cell viability may be obtained even at lower HT doses, as demonstrated by 72 h treatment with 50 μM and 100 μM HT, which significantly diminished the percentage of viable MCF-7 cells [58], and by 100 μM and 150 μM HT-induced reduction in MCF-7 and T47D cell viability after 24 h [47]. Incubating MCF-7 cells with 10–75 μM HT showed an inhibitory effect on E2-induced cell growth, with HT becoming toxic at concentrations ≥ 100 μM. ERα basal activation was induced by 10 μM HT, but (similarly to OLE) when HT was used at concentrations of 10–75 μM, it exhibited no effect on E2-induced ERα activation and E2-modulated ERα expression, while reducing levels of phospho-ERK1/2 (pERK1/2) [45]. As regards invasiveness of ER-positive cells, experimental evidence obtained in MCF-7 and T47D cells confirmed that 50 μM HT elicited effects similar to those recorded for OLE on HGF- and 3-MA-induced cell migration [47].

In human triple negative breast cancer cells MDA-MB-231, HT seemed to lose effectiveness with respect to time, with IC50 values changing from 107.17 μM to 183.65 μM in 72 h [37]. For the same cell line, higher IC50 values (230 μM) were reported for 72 h treatment [61]. On the contrary, another study demonstrated that 100 μM HT promoted MDA-MB-231 cell viability during the first 24 h, becoming ineffective after 48 and 72 h, whereas 250 μM HT became able to reduce cell viability after 72 h treatment, and 400 μM HT significantly diminished cell viability at all the three assayed time points (24, 48, and 72 h) [46]. This piece of data is openly conflicting with reports documenting a loss of MDA-MB-231 viability after 72 h incubation at all tested HT doses (10–100 μM) [58]. The reduction in triple negative breast cell viability may depend on HT acting as a copper chelator, thus perturbating copper homeostasis, as demonstrated in MDA-MB-231 cells by 100 μM HT-mediated increase in the copper chaperone for superoxide dismutase (CCS) after 48 h, and reduction in the subunit II of the complex IV of the mitochondrial respiratory chain cytochrome c oxidase (CcO) after 72 h. Also, HT altered epithelial and mesenchymal markers, and reduced MDA-MB-231 aggressiveness and migration by diminishing copper-dependent Akt phosphorylation. Similar conclusions were drawn for MDA-MB-468 cells [61,170,171]. Other triple negative breast cancer cell lines were identified as particularly resistant towards HT-mediated cytotoxicity, e.g., SUM159 (IC50 = 300 μM) [61]. In naturally HER2-overexpressing human breast cancer cells SKBR3, HT concentrations up to 100 μM failed to elicit a cytotoxic response after 5 days, and only weakly interfered with cell proliferation, but reduced HER2 protein expression after 48 h. However, in engineered HER2-overexpressing MCF-7 cells, 100 μM HT efficaciously triggered apoptosis, diminished cell proliferation, and downregulated HER2 [62].

Other authors suggest that in vitro cytotoxic action of OLE and HT may depend on cell density in culture, hypoxia, and ROS homeostasis. Han et al. observed that incubation with 200 μg/mL OLE and 50 μg/mL HT for 12 h exhibited the most efficient inhibition of cell growth when MCF-7 seeded cell number did not exceed 2 × 10^3^ cell/well in a 96-well plate [42]. Hypoxia increased HT cytotoxicity in MCF-7 cell line, an effect that became evident at 400 μM (vs. 600 μM in normoxic conditions). In hypoxic conditions, 200 μM HT was also able to exert the same actions on oxidative stress response and energy homeostasis as those reported above for normoxic MCF-7 cells [54]. Moreover, in hypoxic MCF-7 cells, 75–200 μM HT reduced the amount of PARP-1, a DNA-binding protein involved in oxidative stress response, but inhibition of PARP-1 activity was achieved only with 200 μM HT [55]. The same concentration of HT accounted for the reduction in the phosphorylated form of kinase mammalian target of rapamycin (mTOR), which in turn produced a reduction in hypoxia inducible factor-1α (HIF-1α), one of the two subunits of the heterodimeric transcription factor HIF-1, ruling the cellular response to hypoxia [55,172,173,174,175]. More importantly, 200 μM HT upregulated the transcription of angiogenic factors adrenomedullin (AM) and VEGF with an HIF-1α-independent mechanism [55]. Treatment of MDA-MB-231 cells with 200 μg/mL olive leaf extract containing 87% OLE for 24 h induced S phase cell cycle arrest and apoptosis by a mechanism that was strongly dependent on OLE-mediated ROS accumulation and downregulation of protein expression of catalase (CAT) and SOD2 [59]. Treatment of MCF-7 cells and another breast cancer cell line generically indicated by the authors simply as “MDA” with 25–100 μM HT for 72 h reduced cell viability in a dose-dependent fashion (mean IC50 ± S.D. = 52 ± 4 μM for of MDA and 58 ± 8 μM for MCF-7), mainly in culture conditions favoring H_2_O_2_ accumulation [56,57].

HT may also have a role in changing the tumor microenvironment. Aged quiescent normal human fibroblasts were able to stimulate MDA-MB-231 proliferation by synthetizing and secreting large amounts of chemokine C-C motif ligand 5 (CCL5), which in turn activated pro-proliferative ERK1/2 and cyclin D1 signalling pattern. Treatment of 100 μM and 200 μM HT prevented CCL5 accumulation in aged quiescent normal human fibroblasts, limiting the proliferation of MDA-MB-231 cells. A similar growth inhibition was also obtained for MCF-7 cells [60].

### 2.5. Liver and Bile Duct Cancers

Hepatocellular carcinoma (HCC) or hepatoma is the most frequent primary liver tumor and the sixth most common cancer worldwide, with a very modest survival rate and a complex management [176].

Results on OLE-mediated effects on hepatoma cells are conflicting. In vitro, 20–80 μM OLE reduced hepatoma Huh7 and HepG2 cell line viability in a dose-dependent manner in 24 h [63]. As determined in HepG2 cells, OLE effects on cell viability are linked to apoptosis induction, mediated by an increase in Bax and cleaved (i.e., activated) caspase-9, caspase-8, caspase-3, and PARP-1 levels, and a decrease in phospho-Akt and Bcl-2 protein levels. After treating HepG2 cells with 50 μM OLE for 24 h, the measured reduction in cell viability seemed related to OLE-mediated accumulation of ROS [63]. However, one report documented the absence of effects on cell viability after treating HepG2 cells with 10^−8^–10^−4^ M OLE for 24 h [64], and another study described no effect on HepG2 cell viability after 48 h incubation with 15–200 μM OLE [65].

The dose-dependent cytotoxic effect of HT on HCC was demonstrated in human hepatoma HepG2 and Hep3B cell lines, with a time-dependent increase in effectiveness. In fact, 80–200 μM HT significantly affected cell metabolic activity after 48 h, whereas a concentration as low as 30 μM was sufficient to reduce the percentage of viable cells in both cell lines after 72 h of incubation [66]. These effects might rely on inhibition of lipogenesis. In fact, in both cell lines’ fatty acid synthase (FASN) activity was consistently inhibited by 200 μM HT (with significant enzymatic inhibition starting with 30 μM and 80 μM HT for HepG2 and Hep3B, respectively), whereas farnesyl diphosphate synthase (FPPS) lipogenic enzyme activity was inhibited only in HepG2 cells [66,177,178]. A further report confirmed the dose- and time-dependent inhibition of cell viability of human HCC cell lines HepG2, Hep3B, and Huh-7, and ascitic fluid-derived cell line SK-HEP-1 for HT doses up to 400 μM, with G2/M cell cycle arrest and inactivation of cell growth-promoting Akt and NF-κB pathways [67,179]. Instead, the role of redox homeostasis in HT-dependent cytotoxicity is less clear in the context of HCC. Cell antioxidant capacities were promoted by 48 h treatment with 80–200 μM HT in HepG2 cells; on the contrary, the same effect was elicited by only 48 h treatment with 30 μM HT in Hep3B cells [66]. However, HT triggers other molecular mechanisms that may influence HCC cell behavior besides affecting cell viability, at least in vitro. Treatment of human hepatoma HepG2 cells with 50–200 μM HT determined an increase in intracellular ionized calcium levels ([Ca^2+^]i) with the contribution of both Ca^2+^ influxes and mobilization of endoplasmic reticulum depots. Anyway, Ca^2+^ dynamics seemed to be unrelated to HT-dependent reduction in cell viability [68], but they might be implicated in changes in the cell secretome [180,181], although definitive proofs are still missing.

Cholangiocarcinoma is the term used to identify rare heterogeneous cancers arising in intrahepatic and extrahepatic bile ducts [182,183]. Instead, gallbladder cancer is the most common biliary tract cancer, characterized by a very unfavorable prognosis [184,185]. Treatment of cholangiocarcinoma cell line KMBC and TFK-1, and gallbladder carcinoma GBC-SD cells with 25–200 μM HT for 24, 48, and 72 h reduced cell proliferation in a time- and dose-dependent manner. A time- (24–72 h) and dose (75–150 μM)-dependent reduction in ERK1/2 phosphorylation was detected in all the three mentioned cell lines. The 72 h incubation of KMBC, TFK-1, and GBC-SD cells with 75 μM and 150 μM HT caused G2/M phase cell cycle arrest, induced apoptosis, augmented the levels of cleaved PARP, Bax, cleaved caspase-3, and cleaved caspase-9, and reduced Bcl-2 levels. Also, treatment of TFK-1 cell xenograft tumor grown in nude BALB/c mice with peritoneal injection of 500 mg/kg/day HT for 3 weeks reduced tumor growth in vivo [69].

### 2.6. Colorectal Cancer

Colorectal cancer, including tumors of the colon and/or rectum, is the third most common cancer and the second most common cancer-related cause of death worldwide. It is characterized by high rates of acquired multidrug resistance, leading to chemotherapy failure, relapse, and development of lethal disease [186,187]. The classical Dukes’ classification distinguishes between “type A” and “type B” tumors, according to the absence or presence of tumor infiltration in extra-rectal tissues, respectively, and designates as “type C” those tumors with regional lymph node metastasis, and as “type C1” and “type C2” those malignancies exhibiting involvement of more distant lymph nodes [188].

In an in vivo model of azoxymethane (AOM)/dextran sulfate sodium (DSS)-induced colorectal cancer in C57BL/6 mice, 50 and 100 mg/kg OLE reduced the incidence of colonic neoplasms, and levels of proliferation regulators NF-κB subunit p65, phosphorylated form of signal transducer and activator of transcription 3 (STAT3), and phospho-Akt, while increasing Bax protein expression. In addition, 100 mg/kg OLE reduced cell proliferation (as confirmed by reduced expression of proliferation marker Ki67) [70,189].

Colorectal carcinoma cell lines may exhibit different sensitivity towards OLE-mediated effects on cell growth. In fact, treatment of colon carcinoma RKO cell line with 20–80 μM OLE diminished cell viability in a dose-dependent fashion [63]. Similarly, 10–100 μM OLE reduced viability of Dukes’ type C colorectal adenocarcinoma SW620 cell line after 72 h incubation, becoming able to increase the rate of apoptosis at the highest concentration tested [71]. On the contrary, 10–50 μM OLE had no effect on the viability of colon adenocarcinoma HT29 cell line, but induced apoptosis only at a concentration of 100 μM [71]. Incubation with 400 μM and 800 μM OLE reduced HT-29 cell viability at all three tested time points (24, 48, and 72 h), inducing G0/G1 phase cell cycle arrest after 24 h treatment. However, only 800 μM OLE was able to promote apoptosis in HT-29 cells after 24 h incubation [72]. Treatment with 400 μM and 800 μM OLE produced a reduction in protein expression of HIF-1α, which was detected after 2 h and persisted up to 48 h. Instead, p53 protein expression was increased only by 48 h incubation with 800 μM OLE [72].

In vitro, the effects of low or nutritionally relevant HT doses are poorly explored, and HT is suspected to interfere with colorectal cancer insurgence acting as a methylation pattern modifying agent. In fact, incubation of human colorectal adenocarcinoma Caco2 cell line with 10 μM HT increased DNA methylation, leading to the repression of the crucial colorectal cancer promoter endothelin receptor type A (EDNRA) [79,190]. Experimental evidence supports a pro-oxidant action of low doses of HT in colorectal cancer cells. Treatment of human colorectal carcinoma HCT116 cells and human Dukes’ type C colorectal adenocarcinoma SW620 cells with 5–20 μM HT for 24 h determined a raise in the apoptotic rates of both cell lines, ruled by a dose-dependent ROS accumulation detected after 4 h of incubation, which in turn was caused by HT direct inhibition of thioredoxin (Trx) reductase 1 (TrxR1), a key player in redox homeostasis [75,191].

At higher concentrations, effects triggered by HT appear different and sometimes poorly reproducible. Treatment of Caco2 and HT-29 cell lines with 100 μM HT for 8 h led to G1 phase cell cycle arrest, whereas extending the treatment for 48 h caused activation of caspase-3. Also, incubation of both cell lines with 150 μM for 8 h produced an increase in apoptosis rate, but HT contributed to the increase in the percentage of necrotic cells only in HT-29 cells [76]. After 24 and 48 h incubation, 6 ppm (≈39 μM) HT was not sufficient to decrease HT-29 cell viability, but after 72 h treatment, there was a significant cytotoxic effect, with IC50 = 12 ppm (≈78 μM). At this concentration, HT elicited an increase in the transcription of tumor suppressor genes encoding for p21^WAF1/Cip1^ and p27^Kip1^, while decreasing the expression of *CCND1* (encoding for cyclin D1) [77,192]. After 24 h, 100 μM HT showed no effect on cell viability of HT-29 cell line. However, 72 h incubation with 100 μM HT efficaciously reduced cell viability of and triggered apoptosis in HT-29 cells [71]. On the contrary, another report demonstrated a dose-dependent increase in the apoptotic rates of HT-29, HCT-116, and LoVo cells as well as Dukes’ type B colorectal carcinoma cell line SW480 for 24 and 48 h treatment with 100–400 μM HT [73]. Further evidence supports HT-dependent reduction in cell viability of and induction of apoptosis in HT-29 cells also at higher concentrations (600–800 μM) [72], and one study documented IC50 = 750 μM for HT29 and HT29–19A cell lines [78].

A more detailed analysis revealed that treatment of HT-29 cells with 400 μM HT for 24 h produced S and G2/M phase cell cycle arrest and induced apoptosis, flanked by apoptosis ruling events: loss of mitochondrial potential, decrease in anti-apoptotic Bcl-2 protein level, reduction in phosphorylation of Bad, increase in pro-apoptotic Bax and Bak, cytochrome c release, and activation of caspase-3 [72,73,126,127]. A similar S phase cell cycle arrest was also observed for 800 μM HT, and induction of apoptosis also took place after 24 h incubation of HT-29 cells with 600 μM and 800 μM HT [72]. A 16 h incubation with 400 μM HT triggered endoplasmic reticulum stress in HT-29 cells, with activation of unfolded protein response, as indicated by the increase in the spliced form of X-box binding protein 1 (XBP-1) mRNA, the upregulation of chaperone 78 kDa glucose-regulated protein (GRP78/Bip) at both mRNA and protein levels, the transient increase (after 2 h) in phosphorylation of PKR-like ER-associated kinase (PERK) and translation initiation factor-2 (eIF2a) (accounting for inhibition of protein synthesis), and time-dependent increase in CHOP protein levels (responsible for ROS production and Bcl-2 downregulation) and NADPH oxidase 4 (NOX4) [73,193,194,195,196]. In the same cellular model, apoptosis seemed regulated by HT-mediated increase in the phosphorylated form of c-jun N-terminal kinase (JNK), which is a central player in establishing cell faith (apoptosis vs. proliferation) [73,197]. Accordingly, the phosphorylated form of JNK target, c-jun, was also increased in HT-29 cells as a response to treatment with HT. Both JNK and c-jun phosphorylations seem to reach a peak after 30 min and 4 h of incubation. In addition, 400 μM HT induced activator protein 1 (AP-1) transcriptional activity (responsible for the expression of cell cycle regulator genes) [73,198,199], promoted a dynamic phosphorylation of ERK1/2 (with two peaks of phosphorylation—after 30 min and 2 h of incubation—both followed by a reduction in phospho-ERK1/2 levels), and abolished Akt phosphorylation. All HT-dependent effects producing apoptosis as a result seemed to be mediated by protein phosphatase 2A (PP2A) [73]. Moreover, 400 μM HT reduced HIF-1α protein levels and augmented the protein levels of proliferation and inflammation modulator peroxisome proliferator-activated receptor γ (PPARγ) after 48 h incubation; 400 μM HT also elicited an increase in p53 levels starting after 16 h and lasting for 48 h [72,164]. Similarly, 800 μM HT had analogous effects on HIF-1α and PPARγ protein expression starting after 24 h of incubation, but did not change p53 levels [72]. Finally, treatment of HT-29 cells with 200 μM HT caused mobilization of endoplasmic reticulum Ca^2+^ depots, but the contribution of this ionic species to HT-induced apoptosis/effects was not explained [73]. In SW620 cells, HT reduced cell viability at all tested concentrations (10–100 μM), with a mechanism related to a decrease in FASN transcription and activity after 72 h exposure. A detailed analysis of the cell cycle revealed that 10–50 μM HT produced S phase cell cycle arrest, with apoptosis induction when HT was used at a concentration of 100 μM [71]. Treatment of HCT116 cell line with 100–300 μM HT and LoVo cell line with 100–400 HT μM for 72 h led to a dose-dependent decrease in cell viability, with IC50 calculated as 92.83 μM and 140.8 μM for HCT116 and LoVo cells, respectively. Cell cycle analysis revealed that 72 h incubation of HCT116 and LoVo cells with 0.0154 mg/mL (≈100 μM) HT and 0.0231 mg/mL (≈150 μM) HT, respectively, caused G2/M cell cycle arrest and promoted apoptosis [74]. As regards the role of ROS in high-dose HT-mediated effects, a report documented a dose-dependent reduction in cell viability of SW480 (mean IC50 ± S.D. = 82 ± 10) and HCT116 (mean IC50 ± S.D. = 55 ± 7) cells treated with 50–100 μM for 72 h, in conditions favoring the accumulation of extracellular H_2_O_2_ [56,57].

### 2.7. Pancreatic Cancer

Pancreatic cancer, or pancreatic ductal adenocarcinoma, arises from the malignant transformation of pancreatic ductal cells, and represents the third leading cause of cancer-related death worldwide, with extremely low 5-year and overall survival rates [200,201].

Up to now, the study of OLE and HT effects in pancreatic cancer in vivo has been limited. In an in vivo orthotopic model of pancreatic cancer, obtained by injecting mouse pancreatic cancer cells Panc02 in C57BL/6 mice, proved that 200 mg/kg HT for 10 days suppressed tumor growth and proliferation. These results seem to depend on the modulation of the tumor microenvironment, since HT was able to reduce the accumulation of myeloid-derived suppressor cells in lymphoid organs, bone marrow, and tumor tissues [81].

Results in vitro underline the diverse sensitivity that cell lines exhibit against OLE- and HT-mediated effects on cell growth. Neither OLE nor HT exhibited a cytotoxic effect on pancreatic ductal adenocarcinoma cell lines BxPC-3 and CFPAC-1 for doses up to 300 μM, but both were able to decrease cell viability of pancreatic ductal adenocarcinoma cell line MIA PaCa-2 (IC50 = 150.1 μM for OLE and 75.1 for HT) after 72 h incubation. Both 200 μM OLE and 100 μM HT produced G2 phase cell cycle arrest after 24 h treatment. The 48 h incubation with the same concentrations of OLE and HT led to caspase-3-mediated apoptosis. The observed effects may be dependent on OLE- and HT-mediated upregulation of AP-1 components c-jun and fos [80]. Similarly, treatment of pancreatic ductal adenocarcinoma cell line PANC-1 with 10 μM, 32 μM, 100 μM, and 320 μM HT for 24, 48, and 72 h reduced cell viability in a dose- and time-dependent fashion; doses as low as 19 μM were able to induce apoptosis (as detected after 24 h), with caspase-9 and Bax upregulation, and MMP-2 and MMP-9 downregulation at mRNA levels after 72 h incubation [82]. Treatment of mouse pancreatic cancer cell line Panc02 with 50 μM, 150 μM, and 200 μM HT for 48 h elicited a dose-dependent inhibition of cell proliferation and induction of apoptosis. Further experimental evidence revealed that 100 μM HT inhibited the expression of phospho-STAT3 and Cyclin D1 after 24 h [81].

### 2.8. Cervical Cancer

Cervical cancer is the fourth most common cancer among women worldwide, and the protagonist of an internationally shared effort towards prevention. Cervical cancer cases may be classified into two major histological subtypes: squamous cell carcinoma and adenocarcinoma [202].

In human cervical adenocarcinoma HeLa cells, 50–100 μM OLE promoted the accumulation of S phase cells with only a modest increase in the percentage of sub-G1 cells, whereas 200 μM OLE was able to induce G2/M cell cycle arrest and apoptosis through the augmented expression of pro-apoptotic Bax protein, the reduction in anti-apoptotic Bcl-2, the release of mitochondrial cytochrome c in the cytosol, and caspase-9 mediated activation of caspase-3. These effects were connected to (I) an increase in the phosphorylated form of apoptosis regulator JNK, which in turn ruled an increase in the phosphorylated forms of cell cycle controller activator protein 1 (AP-1) components c-Jun and ATF-2, and (II) an increase in the levels of tumor suppressors p53 and p21^WAF1/Cip1^ [83]. Another report documented the cytotoxic effect of 100–1200 μg/mL (≈185–2220 μM) OLE in HeLa cells after 24 and 48 h treatments in a dose- and time-dependent manner, with 24 h IC50 = 600 μg/mL and 48 h IC50 = 300 μg/mL. A 50 μg/mL, 100 μg/mL, 200 μg/mL, 400 μg/mL, and 600 μg/mL OLE-dependent increase in the apoptotic cell rate was detected after 48 h incubation [84]. Treatment with 300 μg/mL OLE downregulated invasion-promoting miR-181b-3p, miR-221-3p, radiosensitivity-promoting miR-16-5p, and anti-apoptotic Bcl-2 and Mcl1, while upregulating invasion-inhibiting miR-29a-3p, miR-34a-5p, miR-125-5p and apoptosis-promoter Fas, p53, TNF receptor superfamily member 10b (TNFRSF10B9, also known as TRAIL), and Bid [84,127,203,204,205,206].

### 2.9. Ovarian Cancer

Ovarian cancer is a highly aggressive neoplasm whose complex management (due to molecular and cellular heterogeneity) together with its commonly delayed diagnosis have made it the fifth cause of death in women. The great majority of ovarian cancers are classified as epithelial belonging to the serous subtype [207,208].

Treatment of high-grade ovarian serous adenocarcinoma cell line HEY with 200 μM and 400 μM OLE led to a reduction in cell viability, with accumulation of cells in G2/M phase and induction of apoptosis. A detailed analysis revealed that 400 μM OLE acted as a pro-oxidant agent, increasing oxygen radical generator labile iron pool (LIP) and ROS levels [43]. Similarly, treatment of ovarian serous adenocarcinoma OVCAR-3 cell line with 200 μg/mL olive leaf extract containing 87% OLE for 24 h induced S/G2M phase cell cycle arrest and apoptosis. These effects were dependent on OLE-mediated ROS accumulation and reduction in CAT and SOD2 protein levels [59].

### 2.10. Prostate Cancer

Prostate cancer is the fifth leading cause of cancer-related deaths among men, whose therapeutic options are mainly defined on the basis of a combination of life expectancy, disease stage/risk classification/presence of metastasis, and cancer sensitivity towards androgens [209,210,211].

Treatment of benign prostatic hyperplasia (BPH) epithelial cell line BPH-1 and prostate cancer cell lines LNCaP and DU145 with 100 μM and 500 μM OLE for 72 h determined a reduction in cell viability, probably as a consequence of the reduction in Akt phosphorylation levels, with cells showing signs of necrosis for the highest of the two tested OLE doses. Treatment with 100 μM and 500 μM OLE for 72 h also produced different effects on ROS homeostasis in the three cell lines. In BPH-1, a reduction in ROS was measured, while no effect was recorded for LNCaP cells, and a pro-oxidant activity with a marked ROS increase was exerted by OLE on DU145 cells. Measurement of non-protein thiol groups as a marker of oxidative stress defense revealed that OLE caused a dose-dependent increase in thiol groups in both BPH-1 and LNCaP cells, but only 100 μM OLE augmented thiol groups in DU145 cells; on the contrary, 500 μM OLE diminished thiol groups in this cell line. As regards oxidative damage protecting enzyme heme oxygenase-1 (HO-1), both 100 μM and 500 μM OLE increased HO-1 protein levels in BHP-1 and LNCaP cells, while in DU145 cells, OLE treatment decreased HO-1 enzyme expression. The levels of γ-glutamylcysteine synthetase (γ-GCS) (involved in reduced glutathione synthesis) were not affected by OLE treatment in the three studied cell lines [85,212,213].

ROS homeostasis also seems to play a fundamental role in HT cytotoxic action on prostate cancer cells. Treatment of prostate cancer cell line PC3 with 80 μM HT caused mitochondrial dysfunction and triggered O_2_^•−^-dependent apoptosis [88]. Similarly, 50–150 μM HT reduced cell viability of PC3 and LNCaP cells after 72 h treatment in a dose-dependent fashion (mean IC50 ± S.D. = 103 ± 7 μM and 146 ± 12 μM for PC3 and LNCaP cells, respectively), with a mechanism that seemed dependent on the ability of culture conditions to favor H_2_O_2_ accumulation (quantified in the medium after 24 h incubation with 100 μM HT as mean ± S.D. = 12.0 ± 2.9 and 8.8 ± 3.4 for PC3 and LNCaP cells, respectively) [56,57].

HT action might overcome androgen sensitivity. Incubation of androgen-sensitive LNCaP and castration-resistant C4-2 cell lines with 50–400 μM HT for 48 and 72 h reduced cell viability in a dose-dependent manner, with the IC50 values for LNCaP = 190 μM and 86.9 μM after 48 and 72 h, respectively, and the IC50 values measured for C4–2 = 176 μM and 76.5 μM after 48 and 72 h, respectively. Cell cycle analysis after 48 h incubation with 100–300 μM demonstrated a dose-dependent cellular accumulation in the G1 phase and apoptosis induction, with (I) caspase-3/7 and PARP activation, (II) reduction in Bcl-2, Bcl-XL, cell cycle progression drivers cyclins D1 and E, and CDK2 and CDK4 protein levels, (III) increased Bax protein levels, (IV) reduced NF-κB p65 nuclear localization, (V) reduction in activation levels of cell growth regulators Akt and STAT3, and (VI) decreased androgen receptor protein levels and activity [86,115,214]. The 48 h incubation of human prostate cancer cell lines LNCaP, 22Rv1, and PC-3 with 30–300 μM HT decreased cell viability in a dose-dependent fashion. As indicated by the authors, mean IC50 ± S.E.M. = 41.17 ± 2.79 μM for LNCaP cells, 9.32 ± 0.50 for 22Rv1 cell line, and 28.88 ± 2.25 for PC-3 cells. Treatment of PC-3 cells with 30 μM and 100 μM HT for 24 h reduced the migration rate in a dose-dependent manner. Treatment of 22Rv1 cells with 10 μM HT reduced prostatosphere number and size after 10 days, and phosphorylation of ERK1/2, cAMP response element-binding (CREB) protein, and JNK after 24 h [87].

### 2.11. Osteosarcoma

Osteosarcoma is a primary malignant bone tumor historically known for its extremely low survival rates even in the case of surgical resection/amputation, which have been improved by the introduction of neoadjuvant and adjuvant chemotherapy [215].

Data about OLE cytotoxic effect in vitro are conflicting, and partially dependent on the used cell line and the duration of the treatment. In human 143B osteosarcoma cells, OLE showed a dose-dependent inhibition of proliferation when used at concentrations of 62.5 μM, 125 μM, and 250 μM for 24 h, and 1 μM–250 μM for 48 h. Also, 100 μM OLE induced autophagy after 48 h and exhibited an anti-migratory effect after 60 h incubation [89].

In human MG-63 cells, 3–50 μg/mL (≈5–92.5 μM) OLE for 24 h reduced cell viability in a dose-dependent manner with IC50 = 22 μg/mL ± 3.6 [90]. Another report documented no effect on MG-63 cell viability at OLE concentrations of 50 μM and 100 μM, while a dose-dependent cytotoxic effect was documented for 200 μM and 400 μM OLE after 24, 48, and 72 h incubation, with IC50 = 346 μM for 48 h incubation [91]. Treatment of MG-63 cells with 20 μg/mL (≈37 μM) OLE for 48 h boosted the transcription of autophagy-related genes unc-51, like autophagy activating kinase 1 (ULK1), activating molecule in BECN1-regulated autophagy protein 1 (AMBRA1), and Bcl-2 interacting protein 3 like (BniP3L), as well as protein levels of ubiquitin binding protein p62, suggesting that OLE activity relies on autophagy induction [90,159].

In Saos2 osteosarcoma cell line, 50 μM OLE caused no changes in cell viability after 24, 48, and 72 h incubation. Treatment with 100 μM OLE affected cell viability only after 48 h incubation, causing no detectable effects after 24 and 72 h incubation. Instead, 200 μM OLE started being effective as a cytotoxic agent after 48 h incubation, with effects confirmed after 72 h incubation. On the contrary, 400 μM showed a consistent cytotoxic action at all the assayed time points (24, 48, and 72 h) [91].

### 2.12. Central Nervous System Cancers

Neuroblastomas are clinically heterogeneous embryonal neuroendocrine tumors, driving important research efforts towards the improvement of survival rates for patients with high-risk disease [216,217].

Both OLE and HT exhibit a cytotoxic action against human neuroblastoma cell line SH-SY5Y. After 48 h treatment, IC50 for OLE was 350 μM. OLE induced cell cycle arrest and apoptosis through upregulation of CDK inhibitors p53, p21^WAF1/Cip1^, p15^INK4b^, and p16^INK4a^, together with downregulation of cyclins D1, D2, and D3, and CDK4 and CDK6 [92,115,218,219]. HT was also effective in the reduction in cell viability of SH-SY5Y cells after 72 h incubation, with mean EC50 ± S.D. = 114.02 ± 1.69 μM, inducing apoptosis [93].

Glioma, a neoplastic lesion arising from glial cells, is the most frequent tumor of the central nervous system, and glioblastoma multiforme accounts for the large majority of all gliomas. Glioblastomas are extremely aggressive and difficult-to-treat cancers, with very modest survival expectancy [220,221,222,223].

Treatment of human glioblastoma cell lines A-172 and U-251 with 200 μM and 400 μM OLE for 24 h reduced cell viability in a dose-dependent fashion, triggering apoptosis and suppressing cell migration and invasion abilities with a mechanism involving the increase in Bax, MMP2, MMP9, and phospho-Akt levels, the reduction in Bcl-2 protein levels, and caspase-9 and caspase-3 activation [94]. In glioblastoma cell line T98G, 277.5 μM and 555 μM OLE for 24 h diminished cell viability in a dose-dependent manner. Treatment with 555 μM OLE strongly increased the expression of miRNAs inhibiting tumor growth: miR-181b, miR-137, and Let-7d. On the contrary, 277.5 μM OLE increased Let-7d levels only. Expression of miR-153 was not changed by OLE treatments [95,224,225,226].

### 2.13. Other Solid Tumors

In this section, the most important results about less investigated malignancies are summarized.

Head and neck squamous cell carcinoma is the sixth most common cancer and arises from mucosal epithelium in the oral cavity, pharynx, and larynx [227,228]. Treatment of human cell lines Tu686 and CAL-27 with 50–200 μg/mL OLE (≈92.5–370 μM) significantly reduced cell viability, while no effect was elicited by concentrations ≤ 25 μg/mL (≈46 μM). Consistently, 25 μg/mL did not change the apoptotic rate in the mentioned cell lines, but inhibited epithelial-to-mesenchymal transition (EMT) induced by transforming growth factor-β1 TGF-β1 [96,229]. The effects of OLE on EMT-related proteins were also verified in a in vivo model of 686LN-M2 cell line xenograft in BALB/c nude mice [96].

Among gastric cancers, gastric adenocarcinoma is the most common subtype [230,231]. Treatment of human gastric adenocarcinoma cell line CRL-1739 with 50–500 μM OLE for 24 h reduced cell viability in a dose-dependent fashion, with results becoming significant for values between 200 μM and 500 μM. The authors calculated that IC50 = 42 μM. As demonstrated by incubating the mentioned cell line with 200–500 μM OLE, 24 h exposure elicited the accumulation of ROS and apoptosis [97].

Seminoma is the result of germ cell malignant transformation. It is generally associated with an excellent prognosis, and may involve testicles or extra-gonadal sites [232]. The 48 h incubation of human seminoma cell lines SEM-1 and TCAM-2 with 15–200 μM OLE reduced cell viability in a dose-dependent manner, with IC50 = 140 μM and 50 μM for SEM-1 and TCAM-2, respectively. Treatment of the mentioned cell lines with OLE concentrations corresponding to their respective IC50 values produced apoptosis, the reduction in protein levels of cyclin D1 and nuclear localization of NF-κB, the increase in Bax and p21^WAF1/Cip1^, and impaired cell migratory capacities through downregulation of TGF-β1 [65].

## 3. Effects of OLE and HT on Hematological Malignancies

Hematological malignancies arise from the loss of hematopoietic homeostasis, and may be configured as a large category including both myeloid and lymphatic neoplasms (leukemia, lymphoma, and multiple myeloma) whose incidence is pretty variable at the regional level [233,234]. Only limited experimental evidence supporting OLE and HT preventive and anti-cancer properties is available in such a context (Table 1).

### 3.1. Acute Promyelocytic Leukemia

Acute promyelocytic leukemia is a form of acute leukemia characterized by a chromosomal rearrangement involving PML::RARA fusion [235], making this malignancy sensitive to differentiating agents inducing PML-RARA fusion protein degradation by targeting the RARA or the PML part (all-trans-retinoic acid and arsenic-trioxide, respectively) [236].

In vitro, treatment of promyelocytic leukemia cell line HL-60 with 50–100 μM HT reduced cell viability and triggered apoptosis, as indicated by the cleavage of PARP, the activation of caspase-3, and cytochrome c release [99]. According to another report, 100 μM HT reduced DNA synthesis in HL-60 cells, with promotion of cell differentiation, cell accumulation in G0/G1 phase, and apoptosis of S phase cells [78,100,102]. The detected cytotoxic effect of HT was caused by HT-mediated H_2_O_2_ accumulation in cell culture medium, overcoming HL-60 ability to clear ROS [57,102]. The accumulation of H_2_O_2_ and the extent of apoptosis were inversely correlated with cell density [102]. As hypothesized by the authors, in their experimental setting, the first step of HT-mediated H_2_O_2_ accumulation is the auto-oxidation of HT by O_2_, with production of the corresponding o-quinone and superoxide O_2_^•−^; the process is accelerated by SOD [57]. These findings were confirmed in a study using an HT-rich natural extract of the olive pulp as a source of HT [101]. Effects of HT on cell cycle and induction of apoptosis in replicating cells seemed to be related to an increase in cyclin D3 flanked by a decrease in CDK6 when HT concentration was 100 μM, whereas it was linked to an increase in p21^WAF1/Cip1^ and p27^Kip1^ mRNA and protein levels when HT was used at a concentration of 75 μM. As suggested by the authors, these observations may be explained in the light of cyclin D3-dependent CDK6-mediated phosphorylation of pRB, which in turn causes the release of E2F transcription factor and G1/S transition as well as DNA synthesis. Intriguingly, both p21^WAF1/Cip1^ and p27^Kip1^ are able to inhibit CDK6-mediated pRB phosphorylation and accelerate HL-60 differentiation [100].

### 3.2. Other Hematological Malignancies

Chronic myelogenous leukemia (also known as chronic myeloid leukemia) is a myeloproliferative neoplastic disease characterized by the chromosomal translocation t(9;22)(q34;q11.2), resulting in the presence of the Philadelphia chromosome. The mentioned translocation produces the BCR- ABL1 fusion oncogene, encoding for a constitutively active tyrosine kinase, making leukemia cells sensitive to tyrosine kinase inhibition, with very good 5-year survival rates (90%) [237,238,239].

Incubation of chronic myelogenous leukemia cell line K562 for 4 days with 200 μg/mL (≈370 μM) and 400 μg/mL (≈740 μM) OLE reduced cell density and viability. After 48 h treatment with 200 μg/mL OLE, a significant activation of caspase-1 was measured. Also, 200 μg/mL OLE caused a reduction in Prdx-1 protein level after 8 h of treatment, which was maintained after 24 h [98]. Treatment of K562 cells with concentrations of HT up to 1000 μM produced a dose-dependent reduction in cell viability (EC50 = 147 μM) with concomitant increase in caspase 3/7 activity [103].

Acute monocytic leukemia is the expression formerly utilized to indicate a neoplastic lesion arising from the loss of normal maturation along the monocytic lineage, and is now included in the larger category labeled as acute myeloid leukemia (that is the most frequent leukemia among adults) [240].

THP-1 is probably the most known acute monocytic leukemia cell line. Among all HT doses tested (1–40 μM), only 20 μM affected viability of THP-1 cells after 72 h [104]. Instead, in U937 cells (a human acute myeloid leukemia cell line of pro-monocytic origin) [241], 75 μM and 200 μM HT increased cell death and apoptosis [105].

T-cell acute lymphoblastic leukemia (T-ALL) represents the consequence of the loss of proper regulation of T-cell development, resulting in the accumulation of immature progenitors. With conventional therapies, survival is lower among adult patients vs. pediatric subjects, mainly because of treatment-associated toxicity and higher relapse rates in adults [242,243,244].

Treatment of T-ALL cell line CCRF-CEM with HT concentrations up to 1000 μM produced a dose-dependent reduction in cell viability (EC50 = 338 μM), flanked by the increase in caspase 3/7 activity [103]. Despite being considered as a T-ALL cell line, Jurkat cells are immunophenotypically different from T-ALL cells; thus, results obtained in this cell model retain modest reliability [245]. A study performed using an HT-rich natural extract of the olive pulp as a source of HT demonstrated a dose-dependent reduction in cell viability and induction of apoptosis, together with ROS accumulation [101].

## 4. Discussion

As the literature analysis in Section 2 and Section 3 corroborates, methodological standardization for the study of the effects of natural products may reveal to be challenging, mainly as a consequence of the combination of the multiplicity of molecular patterns triggered and effects elicited by these substances, together with the intrinsic variability of the biological systems (cancer cells and interaction with the surrounding tissue cells) of interest.

**Figure 2:**
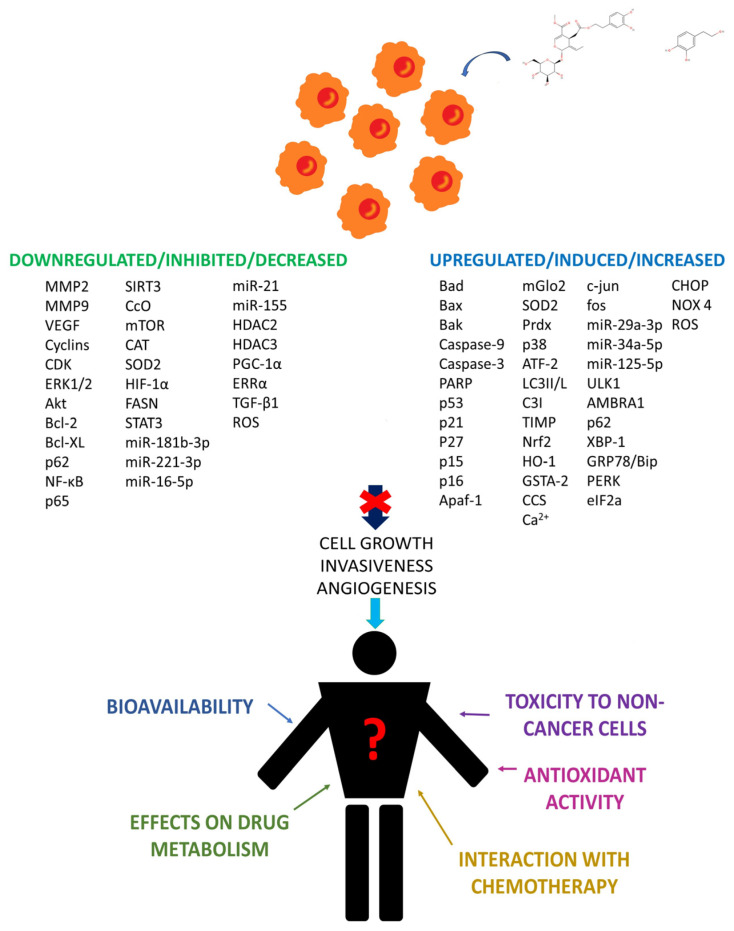
Schematization of molecular in vitro and in vivo effects determining OLE- and HT-mediated suppression of cancer cell growth, and factors influencing OLE and HT cytotoxicity in humans. In the upper left part of the image, mediators whose expression is downregulated and/or activity is suppressed and/or levels are reduced by OLE and HT are indicated. In the upper right part of the figure, pathways induced and mediators upregulated by OLE and HT are reported. OLE and HT induce the activity and/or increase the levels of pro-apoptotic mediators, while inhibiting patterns involved in proliferation and survival. The role of some actors (like autophagy marker p62 and intracellular calcium levels) still needs to be elucidated. ROS function is still poorly understood, since oxidative stress seems involved in OLE- and HT-mediated reduction in cancer cell viability, despite OLE and HT being antioxidants. It is worth noting that these results are not expected to be automatically confirmed in cancer patients, since the role of modest OLE and HT bioavailability, OLE and HT interference with drug metabolism and chemotherapy effect, and OLE and HT toxicity to non-cancer cells have been barely investigated in the context of cancer prevention and cancer treatment. Molecular structures were designed with MolView Copyright© 2014–2023 Herman Bergwerf available at https://molview.org/ (accessed on 13 February 2024); the artwork was made with Microsoft Paint 3D version 6.2310.24037.0 (Microsoft Corporation, Redmond, WA, USA).

The dissertation above seems to validate the use of OLE and HT as cytotoxic agents, with the chance of a further employment of both compounds as microenvironment modulators (OLE- and HT-dependent effects on cancer cell viability and behavior are summarized in Figure 2). However, before extrapolating any conclusion, the reported data should be interpreted and discussed in the light of factors determining the feasibility of such a purpose. In the next paragraphs, we discuss the role of OLE and HT absorption, bioavailability and toxicity to non-cancer cells, OLE and HT interaction with chemotherapy drugs, OLE- and HT-mediated repercussions on drug metabolism, and OLE and HT antioxidant activity in the context of chemotherapy-induced oxidative stress as determinants of the net OLE and HT activity on cancer insurgence and growth in humans. Factors influencing OLE and HT effects in human cancers are reported in Figure 2.

### 4.1. Use of OLE and HT in Clinical Routine: Absorption, Bioavailability, and Safety

Information about OLE and HT absorption through the digestive tract and bioavailability is limited, and often arises from a combination of experimental proofs obtained from humans and animal models.

After ingestion, OLE remains mostly stable in the acid gastric environment, although non-enzymatic hydrolysis may account for an increased amount of HT reaching the small intestine. OLE is poorly absorbed through the small intestine (mainly via diffusion), as demonstrated in rats and humans, enters systemic circulation, undergoes sulphate and glucuronide conjugation and/or enzymatic conversion in HT, and is eliminated in urine mainly as aglycon and glucuronide derivatives [22,26,246,247,248,249,250,251,252,253,254,255,256,257]. In the large intestine, OLE is metabolized by gut microbiota, producing HT [22,26,247,248,256].

After ingestion, HT is more largely absorbed in the intestine by diffusion, is mainly metabolized into glucuronide and sulphate conjugates, and is excreted in urine mostly in its glucuronide conjugated form [22,26,250,251,253,255,257,258,259,260,261,262,263,264]. For both OLE and HT (and their metabolites), the maximum excretion rate is reached in 4 h in humans [251]. All these aspects may represent a potential source of difficulties if the objective is achieving and maintaining pharmacologically relevant concentrations of OLE and HT in plasma after ingestion.

Whenever performing a critical evaluation of OLE and HT availability, data from animal models should be taken into account with caution, since it has been demonstrated that the rate of excretion of HT differs between humans and rats (being more rapid in humans) [262]. Thus, only data related to OLE and HT plasma concentrations in humans are listed in this section. In a group of volunteers, the efficacy of OLE delivery was assayed for liquid and capsule preparations, each containing a lower (64 mg total olive phenols, with 51.1 mg OLE) or a higher (96 mg total, with 76.6 mg OLE) dose. The best performance was offered by higher dose liquid preparation, with a mean peak of plasma OLE ± S.D. = 3.55 ± 2.27 ng/mL and a mean time to peak ± S.D. = 20 ± 12 min, whereas the worst OLE peak plasma values were detected for the lower dose capsule preparation (mean peak ± S.D. = 0.52 ± 0.24 ng/mL, mean time to peak ± S.D. = 40 ± 27 min) [254]. In humans, the assumption of 5 mg HT added to extra virgin olive oil produced a plasma peak of 3.79 ng/mL after 30 min, followed by a rapid decline in HT plasma concentration (minimum reached value < 2 ng/mL after 240 min) [265]. This result matched another report documenting that the consumption of HT-enriched biscuits (containing 5.25 mg HT) determined the appearance of HT metabolites in the volunteers’ plasma between 30 min and 1 h [266]. A similar pharmacokinetic effect was reported in other experimental settings using olive-derived watery supplements as a source of HT [267]. The ingestion of 40 mL of high (366 mg/kg)-phenolic-compound-content olive oil led to a plasma HT concentration peak of ≈15 μM (mean time to peak ± S.D. = 0.91 ± 0.84 h, mean estimated elimination half-life ± S.D. = 3.00 ± 1.46 h), whereas the same amount of low- (2.7 mg/kg) and medium (164 mg/kg)-phenolic-compound-content olive oil produced an HT peak of ≈5 μM or less [268]. The assumption of 25 mL of extra virgin olive oil led to a maximum plasma concentration = 4.4 ng/mL, (time to peak = 0.25 h) [269], and the ingestion of 25 mL of low-phenolic-content (10 mg/kg), moderate-phenolic-content (133 mg/kg), and high-phenolic-content (486 mg/kg) olive oil produce a plasma HT peak of ≈5 nM, 25 nM, and 50 nM, respectively [270].

On the basis of these pieces of data, it becomes evident that cytotoxicity and anti-cancer effects of OLE and HT were recorded at concentrations largely exceeding those reachable with diet/olive oil consumption, and OLE and HT pharmacokinetics does not match the requested treatment duration to exert an anti-proliferative effect. Thus, it is difficult to imagine how OLE and HT may be used as cancer-preventive/treating agents if the route of administration is ingestion. Also, given that both phenols are extensively metabolized and rapidly excreted, the safety and efficacy of other routes of administration (e.g., intravenous) should be assessed in detail.

However, even at high concentrations, OLE and HT seem to be selectively cytotoxic for cancer cells, with no or negligible/minimal effects on non-cancer cells, as demonstrated for embryonic rat cardiomyoblasts H9c2(2-1), human breast epithelial cell line MCF-10A [41,93], nonmalignant human bronchial epithelial BEAS-2B cell line [35], normal colonic cell line CCD-841CoN [74], human normal liver cell line (HL-7702) [67], human normal prostate epithelial cells PWLE2 [86], human bile duct cell line HIBEpiC [69], human fibroblasts WI-38 [90], normal skin fibroblast cell line WS1 [74], human GN61 gingival fibroblasts [271], human lymphocytes [78], human PBMCs [24,25], and normal human fibroblasts [272,273]. Thus, OLE and HT are generally considered safe on the basis of experimental data, despite sporadic reports against the trend, as happened for HT, which proved to be toxic for human non-tumorigenic pancreas cells HPDE [80] and human normal prostate RWPE-1 cells [87]. The evaluation of OLE and HT safety profile in cancer patients is still pending.

### 4.2. Effects of OLE and HT on Drug Action and Metabolism

Some experimental proof has demonstrated that OLE and HT may potentiate the effect of both routinely employed and new potential anti-cancer drugs [274].

In human melanoma cell line A375, the combination of 250 μM OLE with alkylating agent dacarbazine was more effective in reducing cell viability than dacarbazine alone [30]. In female breast cancer patients undergoing neoadjuvant chemotherapy, orally administered 15 mg/kg HT determined a significant decrease in plasma levels of TIMP-1 during treatment with epirubicin and cyclophosphamide [275]. In an in vivo model of tumor xenograft (triple negative MDA-MB-231 cell line) in BALB/c OlaHsd-foxn1 mice, peritoneally injected 50 mg/kg OLE for 4 weeks exhibited a synergistic effect with doxorubicin on inhibition of tumor growth and induction of apoptosis [40]. Combination of paclitaxel with HT reduced MCF-7 and MDA-MB-231 cell viability in vitro, and tumor volume in breast cancer-bearing Sprague–Dawley rats (in vivo injected HT dose = 0.5 mg/kg/day) [58]. In HT-29 and WiDr colorectal cancer cell lines, combination of 10 μM HT and monoclonal anti-epidermal growth factor receptor (EGFR) antibody cetuximab reduced cell growth, both in the presence and in the absence of epidermal growth factor (EGF) stimulation, inducing G1/S and G2/M phase cell cycle arrest [276]. In human osteosarcoma MG-63 cell line, 20 μg/mL (≈37 μM) OLE had an additive effect on anthracycline Adriamycin-induced reduction in cell viability, with a mechanism that did not alter the G2/M phase blockade elicited by Adriamycin [90]. Similarly, in 143B osteosarcoma cells, OLE showed a synergistic, antiproliferative, and anti-migratory effect in combination with estradiol metabolite 2-methoxyestradiol at all OLE concentrations tested (1–250 μM for proliferation, and 100 μM for wound healing assay) [89]. On the contrary, HT did not modify doxorubicin-mediated growth inhibition of human osteosarcoma cells U-2 OS [277]. In neuroblastoma cells T98G, 277.5 μM and 555 μM OLE showed a synergistic effect with alkylating agent temozolomide on cell viability, increasing the expression of miRNAs involved in tumor growth suppression, mainly Let-7d [95].

OLE and HT may also magnify the efficacy of other types of cancer treatment. In nasopharyngeal cancer cell line HNE1 and HONE1, 200 μM OLE enhanced cell radiosensitivity in vitro and in vivo after injection in BALB/C nude mice, with a mechanism involving OLE-dependent removal of HIF-1α hypoxic repression exerted at miR-519d promoter region, upregulation of miR-519d, and miR-519d targeting of DNA damage-regulated protein 1 (PDRG1) [278].

Despite these encouraging results, since both OLE and HT may act as transcriptional regulators and are extensively metabolized by the liver (see sections above), a careful analysis of the effects of both phenols on phase I and phase II enzyme kinetics and expression should be performed before considering the use of OLE and HT during chemotherapeutic treatments. Preliminary data obtained in human liver microsomes point towards OLE-mediated inhibition of CY3A and CYP1A2 activity [279,280]. On the contrary, a study performed in 129/Sv WT and *Ppara*-null mice demonstrated that OLE (ingested with food, thus absorbed through the intestinal wall) stimulated the transcription of cytochrome P450 genes *Cyp1a1*, *Cyp1a2*, *Cyp1b1*, *Cyp3a14*, *Cyp3a25*, *Cyp2c29*, *Cyp2c44*, *Cyp2d22*, and *Cyp2e1* in the liver, with a mechanism mediated by peroxisome proliferator-activated receptor α (PPARα) [281]. Studies defining the effects of OLE and HT on the expression of and interaction with phase I and phase II enzymes are still missing, but they should be considered absolutely necessary in order to determine if OLE and HT are able to modify the metabolism of other drugs.

### 4.3. Use of OLE and HT to Alleviate Cancer Risk Factors and Chemotherapy Toxicity: ROS Homeostasis and Inflammation

Besides their possible synergic/additive actions, OLE and HT might also be seen as useful support agents during cancer treatment. A lot of experimental data in vivo and in vitro have definitively demonstrated the ROS scavenger ability of OLE and HT, which can also act on antioxidant cellular mechanisms restoring ROS homeostasis, including promotion of the increase in reduced glutathione levels (GSH), depletion of lipid peroxidation product malondialdehyde (MDA), intensification of the expression and/or activity of detoxicating enzymes SOD, CAT, glutathione-S-transferase (GST), and glutathione peroxidase (GSH-Px), and nuclear factor E2-related factor 2 (Nrf2) upregulation/transactivation, which in turn regulates the expression of fundamental enzymes protecting cells from oxidative damage, like HO-1 [24].

Radical and non-radical ROS (including hydrogen peroxide H_2_O_2_, superoxide anion radical O_2_^•−^, and hydroxyl radical ^•^OH) may have a pro-tumorigenic effect; they are involved in cancer insurgence determining DNA damage, genomic instability, and interference with signalling and metabolic pathways, enhance cell proliferation through the activation of pro-survival pathways in cancer cells while promoting the reorganization of cellular antioxidant capacities and adaptation to hypoxic conditions, are involved in the development of anti-cancer therapy resistance (through the expansion of cell antioxidant capacities), push the activation of metastasis cellular programs via EMT, and boost immunosuppression and angiogenesis in the cancer microenvironment. However, if ROS concentration overcomes cancer cell defenses against oxidative stress and damage, or cancer cell-produced ROS balance is perturbated, ROS may account for fatal cell damage and trigger apoptosis [130,282,283,284,285,286,287,288].

This may explain the fact that the mechanism of action of some common cancer treatments (e.g., radiation, inorganic compounds, tyrosine kinase inhibitors, monoclonal antibodies, protease inhibitors, pyrimidine analogues, alkylating agents, and anthracyclines) relies on oxidative stress and ROS-dependent apoptosis [286,287,288,289,290,291]. OLE and HT cytotoxic actions themselves in part depend on ROS generation [57,59,63,75,85,88,101,102]; moreover, 200 μM OLE reduced HIF-1α mRNA and protein levels in HNE-1 and HONE-1 nasopharyngeal cancer cell lines [278], and pro-apoptotic OLE concentration corresponding to 100 μM was able to increase ROS production in MDA-MB-231 cell line, with the maximum peak obtained after 4 h incubation [51].

ROS-mediated damage at least in part accounts for chemotherapy-dependent toxicity detected at the tissue level on non-cancer cells [283]. However, OLE and HT have shown an important ability to mitigate the toxicity elicited by chemotherapeutic agents mainly through their largely demonstrated antioxidant and ROS scavenger activity.

In fact, in vivo, 50 mg/kg, 100 mg/kg, and 200 mg/kg OLE showed a dose-dependent antioxidant activity, accounting for amelioration of cisplatin-induced pancreatic, liver, lung, and stomach damage in Spraque–Dawley rats [292,293,294]. In the same in vivo model of cisplatin-induced oxidative stress, 50 mg/kg, 100 mg/kg, and 200 mg/kg OLE improved anemia, thrombocytopenia, and leukopenia [295]. In cyclophosphamide- and epirubicin-induced toxicity in Sprague–Dawley rats, four cycles of 150 mg/kg/week OLE reduced lipid peroxidation and increased the activity of antioxidant enzymes in heart, kidney, and liver [296]. Moreover, in a model of cyclophosphamide-induced immunosuppression in broilers, a solution containing 200 mg/L HT reduced duodenal MDA levels while increasing the activity of antioxidant enzymes [297]. In vitro, 50 μM and 70 μM HT reduced doxorubicin-mediated toxicity in embryonic rat cardiomyoblasts H9c2(2-1) after 48 h incubation. Also, 24 and 48 h incubation with 50 μM HT reduced doxorubicin-dependent intracellular ROS accumulation, increasing SOD2 levels and protecting cells from doxorubicin-induced apoptosis [277].

OLE- and HT-dependent redox homeostasis restoration might represent a potential issue with respect to OLE and HT use at nutritionally relevant concentrations as both anticancer drugs and detoxicating agents (especially during chemotherapy). Low concentrations of both OLE and HT (10 μM) protected HL-60 and PBMCs from H_2_O_2_-induced DNA damage, and lymphocytes from PMA-stimulated monocyte-mediated oxidative DNA damage [298]. This piece of information sounds particularly alarming, since arsenic trioxide (As_2_O_3_) is one of the agents utilized to treat acute promyelocytic leukemia, especially in combinatory first-line therapy, and its mechanism of action relies on ROS increase [289,290,291]. Incubation with 30 μM HT for 48 h was not sufficient to reduce viability of Hep3B cells, but significantly improved cellular antioxidant capacities [66]. Similarly, 5–200 μM HT was ineffective as a cytotoxic reagent, but reduced the level of oxidative stress in MCF-7 cells after 16 h incubation [54]. Erastin is a ferropoptosis inducer that is currently under evaluation as an anti-cancer treatment [299,300]. In ovarian carcinoma HEY cells, 100 μM OLE was not able to affect cell viability, but acted as an antioxidant, decreasing endogenous and erastin-dependent LIP and ROS levels, preventing erastin-dependent ROS accumulation in mytochondria and increasing glutathione peroxidase 4 (GPX4) levels that were reduced by erastin treatment, finally counteracting erastin-induced cell death [43]. It remains to be demonstrated if nutritionally relevant and/or non-cytotoxic concentrations of OLE and HT may offer a survival advantage to neoplastic cells. A similar scenario has been described for other antioxidants (e.g., N-acetyl-L-cysteine, alpha-tocopherol and carotenoids), which showed a pro-tumorigenic action by exerting beneficial effects on cancer cells [282,284,301,302].

It will be fundamental to deepen these pieces of data in order to rule out the chance that OLE and HT may promote cancer growth and metastasis.

Another risk factor for tumorigenesis is inflammation. ROS imbalance itself may drive inflammation, inducing the production of pro-inflammatory cytokines interleukin-1 (IL-1), IL-6, IL-8, and tumor necrosis factor-α (TNF-α) [130,285,286]. Persisting (chronic) or poorly controlled inflammation may directly promote tumorigenesis due to the action of ROS and the recruitment of immune cells triggering the release of growth signals and the activation of tissue repair mechanisms. After cancer insurgence, the production of inflammatory mediators sustained by cancer cells and surrounding actors (tissue macrophages, cancer-associated fibroblast, infiltrating immune cells, and endothelial cells, among the others) contributes to the creation of an environment favoring immune evasion, cell survival, invasion, and angiogenesis. Chemotherapy may also account for an exacerbation of inflammation, whose biological meaning is poorly understood, being recognized as immune-activating as well as a contributor to the failure of the therapeutic regimen [303,304,305]. OLE and HT exhibit an anti-inflammatory activity that has been demonstrated in multiple in vivo and in vitro models, although some experimental variables related to the timing of administration of inflammatory stimuli might have affected the reproducibility of the results, mainly in vitro, and nutritionally relevant concentrations of OLE and HT often showed no anti-inflammatory activity on human peripheral blood mononuclear cells [24,25].

Proof of OLE and HT modulation of inflammation in cancer cells has been obtained mostly from colorectal cancer. In an in vivo model of AOM/DSS-induced colorectal cancer in C57BL/6 mice, 50 mg/kg and 100 mg/kg OLE reduced IL-6, TNF-α, and IFN-γ colon tissue levels, as well as COX-2 levels [70]. Treatment of HCT116 and LoVo cells with 0.0154 mg/mL (≈100 μM) HT and 0.0231 mg/mL (≈150 μM) HT, respectively, for 72 h reduced phosphorylation of NF-κB p65, in turn leading to a reduction in pro-inflammatory cytokines TNF-α and IL-8 at both mRNA and protein levels. HT-dependent anti-inflammatory effect was also mediated by HT-elicited increase in protein levels of PPARγ. In addition, HT acted as a PPARγ agonist, promoting its transcriptional activity [74]. In HT-29 cell line, 100–400 μM reduced TNF-α-induced NF-κB activation in a dose-dependent manner [73]. In in vitro models, the frame appears completely different. The only HT concentrations that elicited a reduction in IL-6 levels were 80 μM for HepG2 and 30 μM for Hep3B. HT doses of 100 μM and 200 μM in HepG2 and 80 μM, 100 μM, and 200 μM in Hep3B cells produced an increase in IL-6 release [66]. Treatment of K562 cells with 100 μM HT produced transcriptional effects including the downregulation of IL-10 receptor and the upregulation of inflammatory mediators IL-6 and IL-8 [103].

OLE and HT may participate in control of cancer-associated and chemotherapy-dependent tissue inflammation by acting on non-tumor cells. As demonstrated in vivo, treatment of BALB/cN mice with 5, 10, and 20 mg/kg OLE suppressed signs of cisplatin-induced renal inflammation, including tissue modulation of TNF-α levels [306]. Four cycles of 150 mg/kg/week OLE in Sprague–Dawley rats reduced serum TNF-α and IL-6 in an experimental model of epirubicin and cyclophosphamide toxicity [296]. In a model of cyclophosphamide-induced immunosuppression in broilers, a solution containing 200 mg/L HT promoted the duodenal expression of anti-inflammatory cytokines IL-4 and IL-10 [297].

It remains to be assessed if such a modulatory activity is able to contribute to the immunosuppressant environment surrounding cancer cells.

## 5. Future Perspectives and Conclusions

From the analysis of the literature reported in this review, it becomes evident that the multifaceted nature of OLE and HT interaction with molecular mediators in cancer cells and non-cancer tissues determines the need for safe strategies to improve OLE and HT bioavailability and delivery, also offering a more stable and highly selective anti-proliferative activity throughout time. Different approaches have been evaluated to reach this objective through the development of OLE and HT derivatives or carrier systems. For example, peracetylated OLE had a stronger anti-proliferative and antioxidant activity than OLE in thyroid and breast cancer in vitro [33,307]; iron oxide nanoparticles coated with glucose and conjugated with OLE showed a notable cytotoxic action against colorectal cancer cell line SW480 [308]; chitosan nanoencapsulation of HT was shown to be unaffected by time-dependent dynamic changes in efficacy observed for free HT in lung and breast cancer cell lines [37]; hydroxytyrosyl dodecyl ether, an HT alkyl ether derivative, had a very strong cytotoxic activity at low concentrations (IC50 = 19.9 ± 4.6 μM) in lung cancer cells in vitro [38]; hydroxytyrosyl oleate, an HT ester, exerted the same effects shown by HT on SH-SY5Y neuroblastoma cells at lower concentrations [93]; HT-loaded poly lactide-co-glycolide-co-polyacrylic acidnanoparticles exhibited cytotoxic and transcriptional regulating effects at lower concentrations (6 ppm) than free HT in colorectal cancer in vitro [77].

Anyway, it would be highly recommendable to address future OLE- and HT-specific research directions towards the exploration of the true effect of nutritionally relevant concentrations of these phenols (or OLE and HT concentrations that can realistically be maintained in plasma) on cancer cells, since even most recent evidence is not able to determine if the administration of OLE and HT in humans could be detrimental in terms of tumor growth and anti-cancer drug metabolism/effects. Up to now, conclusive studies assessing the influence of nutritionally relevant concentrations of OLE and HT on tumor insurgence and expansion, as well as proof of the absence of OLE- and HT-mediated alterations of chemotherapy metabolism, are still missing. Another important reason for concern is represented by OLE and HT context-dependent dual action as both antioxidant and pro-oxidant agents, since such a behavior may alter the efficacy of cancer treatment in unexplored ways. Also, OLE and HT modulatory activity on immune cells deserves further investigation. Currently, neither OLE nor HT could be safely considered as cancer-preventive agents or drugs for combinatory therapies without excluding the possibility that nutritionally relevant concentrations of these compounds might facilitate neoplastic cell expansion or even treatment escape.

## Figures and Tables

**Table 1:** List of experimental models used to study OLE- and HT-dependent cytotoxicity in cancer and hematological malignancies.

Malignancy		Model	[C]	Ref.
Melanoma				
	OLE	UVB-irradiated albino hairless HOS: HR-1 mouse	10 mg/kg 25 mg/kg	[27]
		B16F10 allograft HFD-induced melanoma progression in C57BL/6N mouse	0.02% HFD 0.04% HFD	[28]
		C32	None	[29]
		A375	250, 500 and 800 μM	[30]
		WM266-4	250, 500 and 800 μM	[30]
		M21	250, 500 and 800 μM	[30]
	HT	C32	400 and 1000 μM	[29]
		A375	50–500 μM	[31,32]
		MNT1	None	[31]
		HT-144	50–450 μM	[32]
		M74	50–250 μM	[32]
Thyroid cancer				
	OLE	TPC-1	50–100 μM	[33]
		BCPAP	50–100 μM	[33]
	HT	TPC-1	324–973 μM	[34]
		FB-2	324–973 μM	[34]
		WRO	65–973 μM	[34]
Lung cancer				
	OLE	A549	50 and 150 μM	[35]
		H1299	50–200 μM	[36]
	HT	A549	147–230.60 μM	[37,38]
Malignant pleural mesothelioma				
	OLE	REN	25 μg/mL	[39]
Breast cancer				
	OLE	Tumor xenograft (MDA-MB-231) in BALB/c OlaHsd-foxn1 mouse	50 mg/kg	[40]
	OLE	MCF-7	10–1100 μM	[41,42,43,44,45,46,47,48,49]
		MDA-MB-231	12.5–500 μM	[46,50,51]
		MDA-MB-468	500 μM	[50]
		MDA (unknown)	200 μg/mL	[52]
		T47D	150 μM	[47]
	HT	Dimethylbenz[*α*]anthracene-induced mammary tumors in Sprague–Dawley rat	0.5 mg/kg	[53]
		MCF-7	10–600 μM	[42,45,46,47,54,55,56,57,58]
		MDA-MB-231	10–400 μM	[37,46,58,59,60,61]
		MDA-MB-468	100–300 μM	[61]
		SKBR3	100 μM	[62]
		MDA (unknown)	25–100 μM	[56,57]
		SUM159	300 μM	[61]
		T47D	100–150 μM	[47]
Hepatocellular carcinoma				
	OLE	Huh7	20–80 μM	[63]
		HepG2	20–80 μM	[63]
			None	[64,65]
	HT	HepG2	30–400 μM	[66,67,68]
		Hep3B	30–400 μM	[66,67]
		SK-HEP-1	200–400 μM	[67]
		Huh7	200–400 μM	[67]
Cholangiocarcinoma				
	HT	KMBC	25–200 μM	[69]
		TFK-1	25–200 μM	[69]
		TFK-1 xeonograft in BALB/c mouse	500 mg/kg	[69]
Gallbladder carcinoma				
	HT	GBC-SD	25–200 μM	[69]
Colorectal cancer	OLE	AOM/DSS-induced colorectal cancer in C57BL/6 mouse	50 and 100 mg/kg	[70]
		RKO	20–80 μM	[63]
		HT-29	100, 400 and 800 μM	[71,72]
		SW620	10–100 μM	[71]
	HT	SW480	100–400 μM	[56,57,73]
		HCT116	5–300 μM	[56,57,73,74,75]
		HT-29	100–800 μM	[71,72,73,76,77,78]
		SW620	5–100 μM	[71,75]
		Caco-2	10 *, 100 and 150 μM	[76,79]
		LoVo	100–400 μM	[73,74]
Pancreatic cancer				
	OLE	BxPC-3	None	[80]
		CFPAC-1	None	[80]
		MIA PaCa-2	150.1 μM	[80]
	HT	Orthotopic model of pancreatic cancer (Panc02) in C57BL/6 mouse	200 mg/kg	[81]
		BxPC-3	None	[80]
		CFPAC-1	None	[80]
		MIA PaCa-2	75.1 μM	[80]
		PANC-1	10, 32, 100, and 320 μM	[82]
		Panc02	50, 150, and 200 μM	[81]
Cervical cancer				
	OLE	HeLa	50–2220 μM	[83,84]
Ovarian cancer				
	OLE	HEY	200 and 400 μM	[43]
		OVCAR3	200 μg/mL	[59]
Prostate cancer				
	OLE	LNCaP	100 and 500 μM	[85]
		DU145	100 and 500 μM	[85]
	HT	LNCap	30–400 μM	[56,57,86,87]
		C4–2	50–400 μM	[86]
		PC3	30–300 μM	[56,57,88]
		22Rv1	30–300 μM	[87]
Osteosarcoma				
	OLE	143B	1–250 μM	[89]
		MG-63	5–400 μM	[90,91]
		Saos2	100, 200, and 400 μM	[91]
Neuroblastoma				
	OLE	SH-SY5Y	350 μM	[92]
	HT	SH-SY5Y	25–200 μM	[93]
Glioma				
	OLE	A-172	200 and 400 μM	[94]
		U-251	200 and 400 μM	[94]
		T-98G	277.5 and 555 μM	[95]
Head and neck squamous cell carcinoma				
	OLE	Tu686	50–200 μg/mL	[96]
		CAL-27	50–200 μg/mL	[96]
		Tumor xenograft (686LN-M2) in BALB/c nude mouse	1200 μg/mL	[96]
Gastric adenocarcinoma				
	OLE	CRL-1739	50–500 μM	[97]
Seminoma				
	OLE	SEM-1	15–200 μM	[65]
		TCAM-2	15–200 μM	[65]
Hematological malignancies				
	OLE	K562	200 and 400 μg/mL	[98]
	HT	HL-60	50–100 μM	[57,78,99,100,101,102]
		K562	100–1000 μM	[103]
		THP-1	20 μM	[104]
		U937	75 and 200 μM	[105]
		CCRF-CEM	50–1000 μM	[103]
		Jurkat	10–100 μg/mL ^§^	[101]

OLE, oleuropein; HT, hydroxytyrosol; Ref., reference(s); [C], cytotoxic concentrations reported in the cited literature; HFD, high-fat diet; 0.02% HFD, HFD containing 0.02% OLE; 0.04% HFD, HFD containing 0.04% OLE; AOM, azoxymethane; DSS, dextran sulfate sodium; *, non-cytotoxic but cell viability-affecting effects; §, olive pulp extract with a 70% content of phenols, of which 70% is represented by HT.
